# Targeted inhibition of SIRT6 via engineered exosomes impairs tumorigenesis and metastasis in prostate cancer

**DOI:** 10.7150/thno.53886

**Published:** 2021-04-26

**Authors:** Qing Han, Qian Rueben Xie, Fan Li, Yirui Cheng, Tingyu Wu, Yanshuang Zhang, Xin Lu, Alice S.T. Wong, Jianjun Sha, Weiliang Xia

**Affiliations:** 1 State Key Laboratory of Oncogenes and Related Genes, Ren Ji Hospital, School of Medicine and School of Biomedical Engineering, Shanghai Jiao Tong University, Shanghai, China.; 2 Department of Urology, Ren Ji Hospital, School of Medicine, Shanghai Jiao Tong University, Shanghai, China.; 3 School of Biological Sciences, University of Hong Kong, Pokfulam Road, Hong Kong.

**Keywords:** SIRT6, castration-resistant prostate cancer, Notch pathway, therapy, engineered exosomes

## Abstract

The treatment for metastatic castration-resistant prostate cancer patients remains a great challenge in the clinic and continuously demands discoveries of new targets and therapies. Here, we assess the function and therapeutic value of SIRT6 in metastatic castration-resistant prostate cancer.

**Methods:** The expression of SIRT6 was examined in prostate cancer tissue microarray by immunohistochemistry staining. The functions of SIRT6 and underlying mechanisms were elucidated by *in vitro* and *in vivo* experiments. We also developed an efficient method to silence SIRT6 by aptamer-modified exosomes carrying small interfering RNA and tested the therapeutic effect in the xenograft mice models.

**Results:** SIRT6 expression is positively correlated with prostate cancer progression. Loss of SIRT6 significantly suppressed proliferation and metastasis of prostate cancer cell lines both *in vitro* and *in vivo*. SIRT6-driven prostate cancer displays activation of multiple cancer-related signaling pathways, especially the Notch pathway. Silencing SIRT6 by siRNA delivered through engineered exosomes inhibited tumor growth and metastasis.

**Conclusions:** SIRT6 is identified as a driver and therapeutic target for metastatic prostate cancer in our findings, and inhibition of SIRT6 by engineered exosomes can serve as a promising therapeutic tool for clinical application.

## Introduction

Prostate cancer is one of the most prevalent malignancies among men worldwide, with approximately 1.3 million new cases annually 1. In China, its incidence has an increasing trend (2.6%/year) in recent 5 years. Most of the early stage localized prostate cancer can be cured by prostatectomy or radiation therapy, but the treatment for metastatic disease is always hampered by the acquired resistance 2. Prostate cancer arises by the driving of androgens, and the androgen deprivation therapy (ADT) to target the androgen receptor (AR) axis is the mainstay of treatments for metastatic prostate cancer. ADT is effective initially, but most patients inevitably develop into lethal metastatic castration-resistant prostate cancer (mCRPC) within 2-3 years. For instance, the next generation anti-androgen agents such as abiraterone and enzalutamide have been highly effective in reducing AR signaling activity, but the disease eventually progresses and results in mean survival time of only 16-18 months 3, 4. Thus, further understanding of additional molecular alterations related with prostate cancer progression is critical to the development of new targets and therapies to prolong the survival of patients with CRPC.

Sirtuins belong to a highly conserved family of NAD^+^-dependent histone deacetylase, involved in a wide variety of cellular functions such as aging, metabolism, stress response, and energy homeostasis 5, 6. Mammalian Sirtuins have seven family members (SIRT1-7), which differ in cellular compartment localization, catalytic activities and biological functions 7. SIRT6 is mainly localized in the nucleus and conservatively responsible for deacetylation of histone H3 N-acetyl-lysine 9 (H3K9Ac) and histone H3 N-acetyl-lysine 56 (H3K56Ac) to modulate the gene transcription 8, 9, 10. SIRT6 has two additional enzymatic functions, namely, defatty-acylation and ADP-ribosylation 11. SIRT6 is canonically associated with tumor development, and was originally identified as a tumor suppressor 12. Surprisingly SIRT6 was also found to be overexpressed in tumor vs. non-tumor tissues of prostate cancer and implicated an oncogenic role in prostate cancer progression in our previous study 13. Such tumor promoting role has also been evidenced in other cancer types 14, 15. However, detailed studies on the oncogenic role of SIRT6 in prostate cancer and potential applications by modulating SIRT6 are still lacking.

Engineered exosomes are promising and efficient drug delivery systems in the precision nanomedicine field 16, 17, and their utility in clinical trials is promising. Exosomes are 50-150 nm sized, membrane-bound nanovesicles secreted by almost all cells 18. Owing to their favorable safety profile and long circulating half-life, exosomes have spurred a wide interest as delivery systems to transport small interfering RNA (siRNA) or chemical drugs for various therapies 19-21. Furthermore, the surface of exosomes can be modified with desired ligands like aptamer or antibody to guide them to targeted sites 22, 23. Aptamers are short single-stranded oligonucleotides or peptides with specific three-dimensional structures to mimic the functions of antibodies with the added benefit of easy chemical synthesis and modification. The E3 aptamer has been shown to be recognized and internalized by prostate cancer cells rather than non-cancerous epithelial cells 24. Considering that there is no SIRT6 specific inhibitor available, we adopted aptamer-modified exosomes carrying siRNA to knock down SIRT6 in our study.

Herein, we investigated the role and underlying mechanisms of SIRT6 in prostate cancer progression. We then tested the effect of inhibition of SIRT6 by using the aptamer-modified exosomes carrying the SIRT6 siRNA in subcutaneously and orthotopically xenografted tumor models. Our study thus provides preclinical evidence for SIRT6 as a novel therapeutic target in high-grade prostate cancer.

## Materials and Methods

### Clinical human prostate cancer tissue and tissue microarray

**Figure 1:**
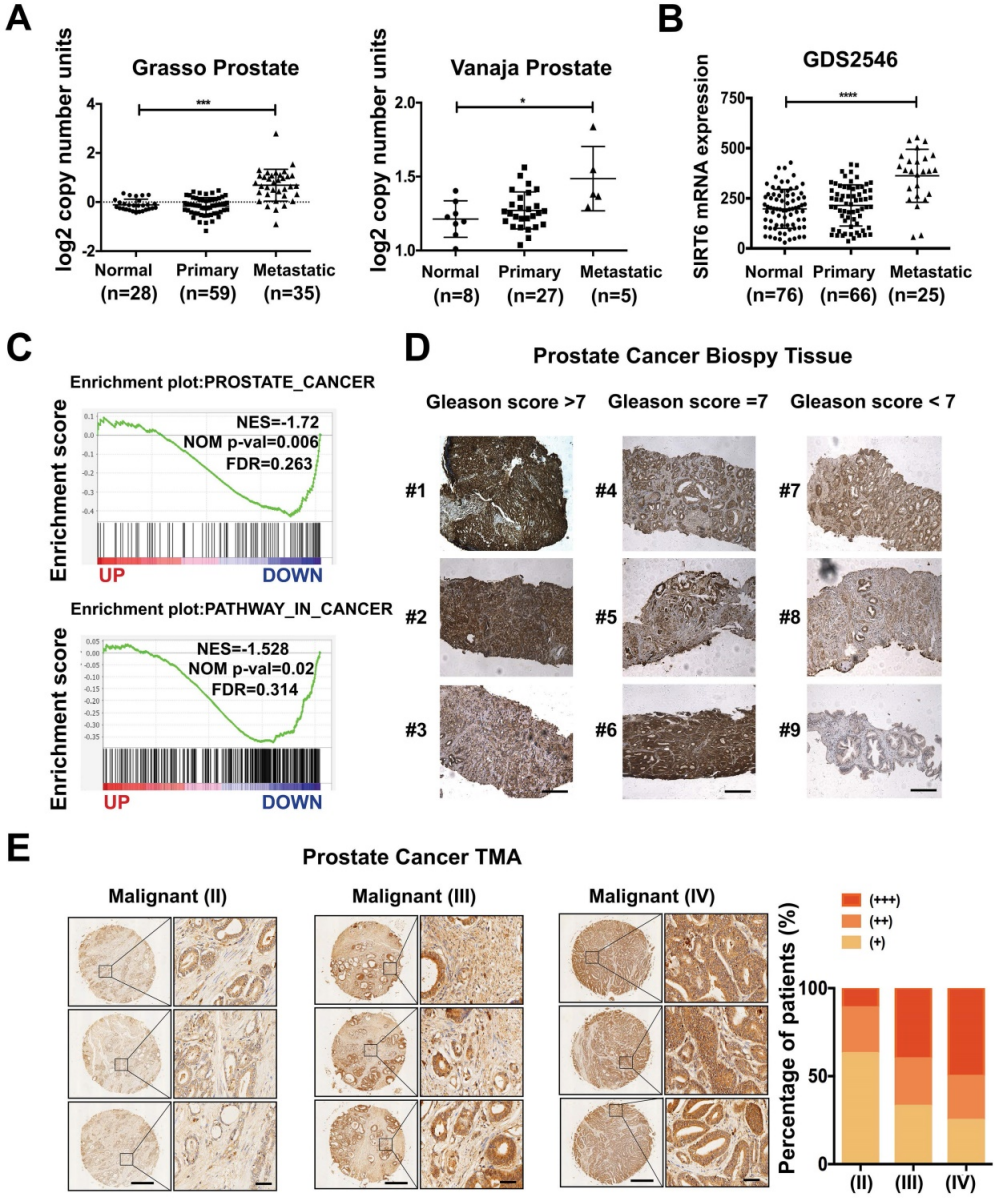
** SIRT6 expression is positively correlated with prostate cancer progression. (A)** Copy number of SIRT6 in normal prostate tissue, primary prostate cancer, and metastasis prostate cancer samples from selected Oncomine database (Grasso Prostate cohort, n=122; Vanaja Prostate cohort, n=40). **(B)** Relative mRNA expression of SIRT6 in GEO database (GDS2546, n=167). **(C)** Enrichment plots of Prostate cancer related gene signatures according to SIRT6 mRNA levels and Enrichment plots of cancer-related pathways dependent on SIRT6 expression in TCGA (PanCancer Atlas) dataset.** (D)** Immunohistochemical staining (IHC) of SIRT6 on prostate cancer biopsy samples grouped by Gleason score. Scale bars, 500 µm. **(E)** Representative IHC of SIRT6 expression in a prostate cancer tissue array. Scale bars, 500 µm and enlarged scale bars, 50 µm (Data are expressed as means ± SEM, Student's t-test, *P<0.05, ***P<0.001, ****P<0.0001).

Prostate cancer biopsy samples (patients n=13) in Figure 1D were obtained from Xiaoshan First People's Hospital 25, and the Prostate cancer biopsy samples in Supplementary Figure 1A (patients n=14) were collected from Department of Urology, Ren Ji Hospital. The tissue microarray (TMA) section for SIRT6 evaluation in Figure 1E was obtained from the Company Avilabio.com (# Pro721), including normal prostate (n=6) and prostate cancer tissue (patients n=18) covering low to high stage prostate cancer (Stage II to IV). SIRT6 staining intensity of TMA was scored into extremely positive (+++), strongly positive (++), positive (+) categories. The study was approved by the Office of the Research Ethics, School of Biomedical Engineering, Shanghai Jiao Tong University.

### Culture of prostate cancer cell lines

Human prostate cancer cell lines C42B and DU145 were purchased from the Cell Resource of Shanghai Institute of Biological Sciences, Chinese Academy of Sciences. PC3 and PC3M-Luc cell lines were kindly provided by Dr. Wei-Qiang Gao (Clinical Stem Cell Center, Ren Ji Hospital). All cell lines were cultured in Dulbecco's modified Eagle's medium (DMEM, Hyclone) supplemented with 10% heat-inactivated fetal bovine serum (FBS, Gemini) and 100×L-Glutamine at 37 °C in a humidified incubator under 95% air and 5% CO_2_.

### Construction of lentivirus vectors and stable transfected cell

Human SIRT6 cDNA construct was cloned into pCDH-CMV-MCS-EF1-copGFP backbone (Addgene plasmid: #72265) to construct plasmid for lentivirus production. HEK293T cells were transfected with empty vector or the SIRT6-overexpression plasmid, accompanied by pLP-VSVG / pCAG-dR8.9 plasmids. Mature SIRT6 overexpression lentivirus and control lentivirus were obtained by ultracentrifugation. SIRT6 shRNA lentiviruses were produced by Hanbio Biotechnology Co. Ltd. (Shanghai, China). Cells were transfected with lentivirus in 24-well plates at 60% confluency, and then stably transfected cells were selected by flow cytometry.

### Quantitative real-time PCR

Total RNA was extracted from cells by RNAiso Plus (Takara, China) and stored at -80 °C. RNA was reverse-transcribed to cDNA by PrimeScript RT reagent kit (Takara, China), and then quantitative real-time PCR was used to determine the expression of mRNA levels by SYBR Premix Ex Taq (Takara, China).

### Colony formation assay and soft agar assay

Cells transfected with either control lentivirus or SIRT6-OE lentivirus were seeded in 6-well plates about 500-1000 cells per well and cultured for two weeks to measure clonogenic ability. The cells were fixed with 4% paraformaldehyde for 20 min, and stained with 0.01% crystal violet solution. The colonies were imaged and then quantified based on percentage of colony area per well using ImageJ software. Soft agar assay was performed in a six-well culture plate coated with 2 ml bottom agar mixture. After the bottom layer was solidified, 5000 cells mixed with 2 ml top agar medium were added. The plate was incubated at 37 °C for about 2-3 weeks, and then the colonies were counted.

### Migration assay

The cells were pre-cultured in serum-free medium for 24 h, then harvested and resuspended in serum-free medium, and were plated on the top of a chamber of an 8 μm-pore membrane (Corning, 356231). Medium supplemented with 10% FBS (800 μL) was added to the bottom of the well. After 24 h, the inserts were washed with PBS and the cells were fixed with 4% paraformaldehyde for 15 min and stained with 0.1% crystal violet for about half an hour. Migration ability was estimated as the number of migrated cells.

### Gene Set Enrichment Analysis (GSEA)

**Figure 2:**
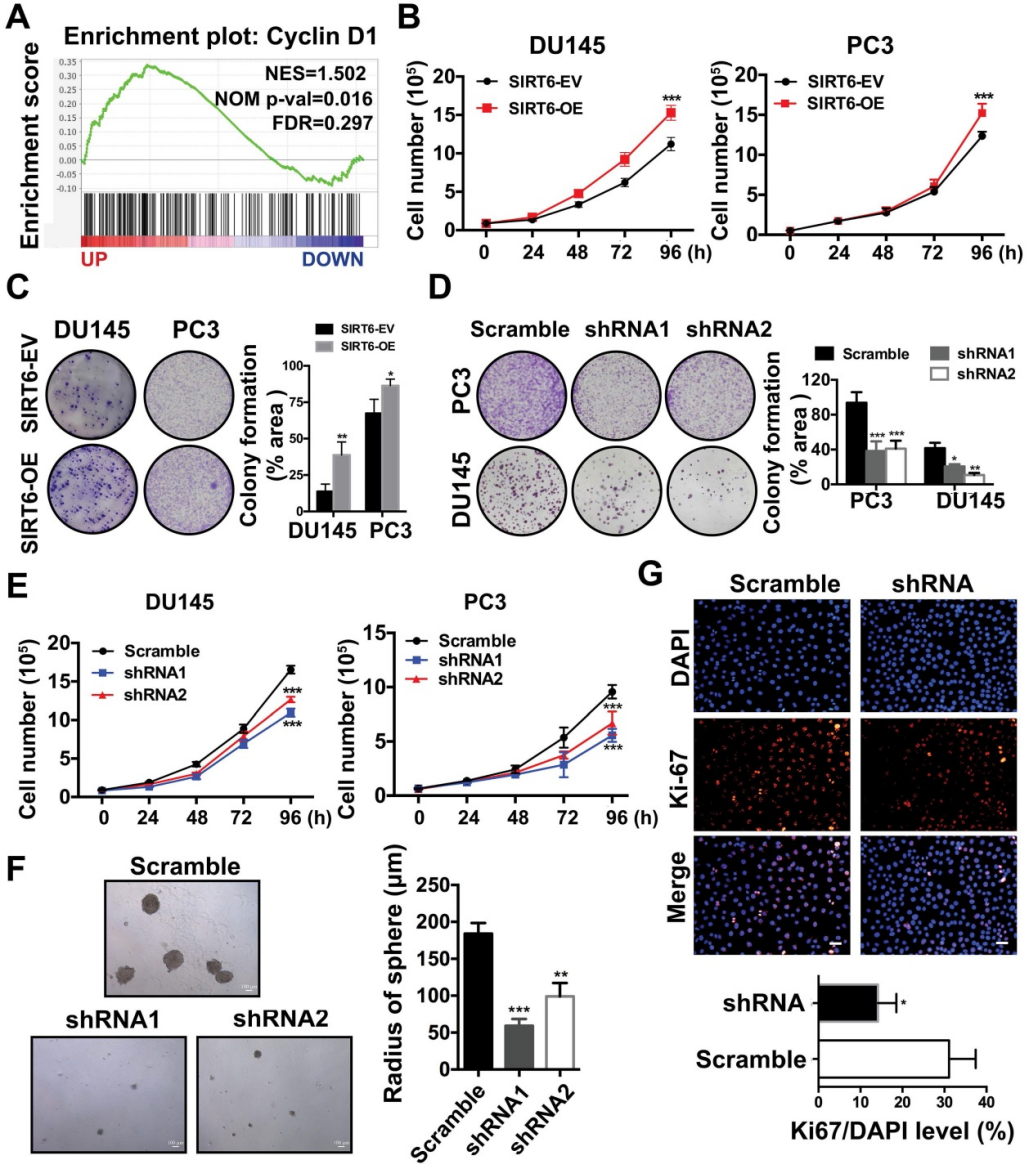
** SIRT6 is essential for the growth of prostate cancer cells. (A)** Enrichment plot of Cyclin D1 according to SIRT6 expression in Metastatic Prostate Cancer (SU2C/PCF Dream Team, PNAS 2019) dataset. **(B)** Proliferation assay in SIRT6 stable overexpression (SIRT6-OE) and control (SIRT6-EV) cancer cells.** (C)-(D)** Colony formation in different prostate cancer cells (DU145 and PC3) with SIRT6 being either up- or down-regulated. **(E)** Proliferation of prostate cancer cells stably transfected with scramble sequence or shSIRT6. **(F)** Soft agar assay in C42B cells stably transfected with scramble sequence or shSIRT6. **(G)** IF staining of Ki67 (red) and DAPI (blue, nucleus) in C42B cells stably transfected with scramble sequence or shSIRT6. Scale bars, 50 µm. The data were represented as mean and SEM from three independent experiments. * P<0.05, ** P<0.01, *** P<0.001.

Gene Set Enrichment Analysis was performed using the GSEA software and the RNA-seq datasets were derived from cBioportal database (http://www.cbioportal.org/datasets), in which three datasets were used. The GSEA results in Figure 1C were analyzed from TCGA (PanCancer Atlas) dataset and the GSEA results in Figure 2A were analyzed from Metastatic Prostate Cancer (SU2C/PCF Dream Team, PNAS 2019) dataset. The signaling pathway of KEGG datasets was ranked by the expression of SIRT6 in Metastatic Prostate Cancer (SU2C/PCF Dream Team, Cell 2015) following the official user guide of GSEA, and then the top 8 signaling pathways were shown in Figure 5E.

### Immunohistochemistry (IHC) and immunofluorescence microscopy (IF)

For immunofluorescence microscopy, cells were plated on cover slides and fixed in 4% paraformaldehyde for 15 min. The fixed cells were permeabilized on 0.5% Triton X-100 for 30 min, and blocked with 10% goat serum for another 30 min, then incubated with Vimentin (Santa Cruzs), Ki67 (Proteintech) overnight at 4 °C. After extensively washing with PBS, secondary antibody, the donkey anti-goat IgG conjugated with Alexa Flour 594 (ThermoFisher, A-11058), was applied to the slides for 1 h. After three times washing, cell nuclei were stained with DAPI (Sigma) for 5 min. The slides were photographed by confocal microscopy.

For IHC, paraffin-fixed tissues were deparaffinized, rehydrated and treated with 0.01 M sodium citrate for antigen retrieval. Endogenous peroxidase activity was blocked by 0.3% (v/v) hydrogen peroxide and then incubated with 0.3% Triton X-100 for 15 min. After blocking with 10 % goat serum for 1 h. The tissues were incubated with primary antibodies at 4 °C overnight. After extensive washing with PBS, tissues were incubated with secondary antibodies (Abcam, ab6884) for 1 h. Diaminobenzidine hydrogen peroxide was used to show staining signaling, then the sections were counterstained with hematoxylin.

### Exosome isolation and purification

Exosomes were collected from HEK293T cell line. HEK293T cells were grown in 15 cm^2^ culture dishes with DMEM medium supplemented with 10% fetal bovine serum (depleted of exosomes). The conditioned media were collected and centrifuged at 500 g for 5 min to remove cell debris. The supernatant was centrifuged at 10,000 g for 30 min and then centrifuged at 100,000 g for 60 min. The pellets were washed with PBS and centrifuged at 100,000 g for 60 min. The final pellets were resuspended with PBS and stored at -80 °C until used.

### Electroporation loading of exosomes with siRNA

Electroporation loading of exosomes with siRNA was conducted based on a previous study 26. Briefly, exosomes were mixed with siRNA in 1:1 (wt/wt) ratio, diluted to 0.5 mg/ml in electroporation buffer. The mixtures were added into ice-cold 0.4 mm cuvettes and electroporated at 400 mV and 125 μF capacitance (pulse time 10-15 ms) in the Gene Pulser Xcell (BioRad, Hercules, CA).

### Conjugation of E3-Aptamer to Maleimide-PEG-Cholesterol

The conjugation was performed by the thiol-maleimide cross-linking reaction. Before the conjugation, C3-thiol-modified E3-Aptamer (GENEWIZ, China) was deprotected by 500 mM TCEP (pH 6.5) to produce Aptamer-SH 27, 28. The deprotected aptamer was then linked to maleimide-PEG-cholesterol at 4 °C overnight followed by dialysis to purify the Aptamer-PEG-cholesterol conjugation.

### *In vivo* xenograft assay

For the subcutaneously xenografted tumor models, 10^6^ prostate cancer cells resuspended in 100 μL PBS were injected into the flank regions of 6-week-old male BALB/c nude mice. Both tumor volume and weight of mice were measured twice a week. For orthotopic implantation model, 10^6^ cells stably expressing luciferase were injected into the anterior lobes of the prostate of 7-week-old male BALB/c nude mice with 31 G insulin syringe. The body weight was measured twice a week. After six weeks, the bioluminescent signal induced by intraperitoneal injection with 150 mg/kg D-luciferin (Synchem, Germany) was detected by Berthold Imaging System (Berthold, Germany). All studies were approved by the Institutional Animal Care and Use Committee, Shanghai Jiao Tong University, and all animals were treated according to the institutional guidelines.

### Statistical analysis

All statistical analyses were performed using the GraphPad Prism software. The data were presented as means ± SEM. The paired or unpaired Student's t-test or ANOVA was used to calculate the statistical significance between two groups. The significance level was set at p<0.05.

## Results

### SIRT6 expression is positively correlated with prostate cancer progression

To determine the role of SIRT6 in prostate cancer, we analyzed the copy number of SIRT6 in the prostate cancer cohorts from the Oncomine database and observed that SIRT6 was highly amplified in prostate cancer tissue samples, especially in metastatic prostate cancer tissue samples (Figure 1A). The copy number of SIRT6 in metastatic prostate cancer tissues (n=35) was much higher than normal tissues (n=28) and primary site tissues (n=59) in the Grasso Prostate dataset containing 122 human clinical specimens. Similar results were also found in another independent dataset, the Vanaja prostate. Consistently, microarray analysis of prostate cancer profiles (GDS2546) in GEO datasets confirmed that SIRT6 mRNA level in metastasis site samples was remarkably increased compared with the normal or primary site tissues (Figure 1B). Furthermore, the gene set enrichment analysis (GSEA) plots for SIRT6 in TCGA (PanCancer Atlas) also revealed that high expression of prostate cancer-related genes and activation of the cancer-related pathways were accompanied with the elevated expression of SIRT6 (Figure 1C), indicating that SIRT6 might have a positive effect on prostate cancer progression. To validate our findings, we examined the SIRT6 expression by immunoblots in benign human prostate tissue (n=7) and prostate cancer tissue (n=7), and found higher SIRT6 levels in prostate cancer tissues than those in normal prostate tissues (Figure S1A). Then we collected another 13 human biopsies of prostate cancer with different Gleason grade scores to assess SIRT6 protein levels. The IHC analysis showed significantly elevated levels of SIRT6 in prostate cancer tissues with high Gleason scores (Figure 1D). Further, we expanded the prostate cancer tissue number by performing IHC staining of SIRT6 in prostate cancer tissue microarray which was categorized by Stage score. The microarray IHC analysis also verified that the levels of SIRT6 significantly increased in high stage prostate cancer tissues (Figure 1E).

Taken together, these results indicated that higher levels of SIRT6 were associated with unfavorable progression in prostate cancer.

### SIRT6 is essential for the growth of prostate cancer cells

Next, we explored the potential function of elevated SIRT6 expression in prostate cancer. Our previous study revealed that SIRT6 was highly expressed in most prostate cancer cell lines 13. Then we chose a panel of prostate cancer cell lines including PC3, DU145 and C42B for further experiments. We generated stable lines of DU145, PC3 and C42B that overexpressed SIRT6 (C42B-OE, PC3-OE and DU145-OE). Likewise, we screened C42B-shSIRT6, PC3-shSIRT6 and DU145-shSIRT6 cell lines by using lentivirus containing two specific SIRT6-targeting shRNA sequences. Western blot verified the SIRT6 overexpression and knockdown effects in these cell lines (Figure S2A, B). The GSEA plots in a Metastatic Prostate Cancer dataset from cBioportal showed that SIRT6 was positively correlated with the cell cycle-related gene Cyclin D1 (Figure 2A), indicating SIRT6 may promote prostate cancer proliferation. Cell proliferation and colony formation were evaluated in DU145 and PC3. Overexpression of SIRT6 markedly enhanced cell proliferation and colony formation (Figure 2B, C), while loss of SIRT6 resulted in a significantly decrease in colony formation and cell proliferation (Figure 2D, 2E). Silencing SIRT6 also reduced the growth of C42B as assessed by tumor sphere assays (Figure 2F) and immunofluorescence staining of Ki67 (Figure 2G).

### SIRT6 induces a metastasis-promoting phenotype in prostate cancer cells

**Figure 3:**
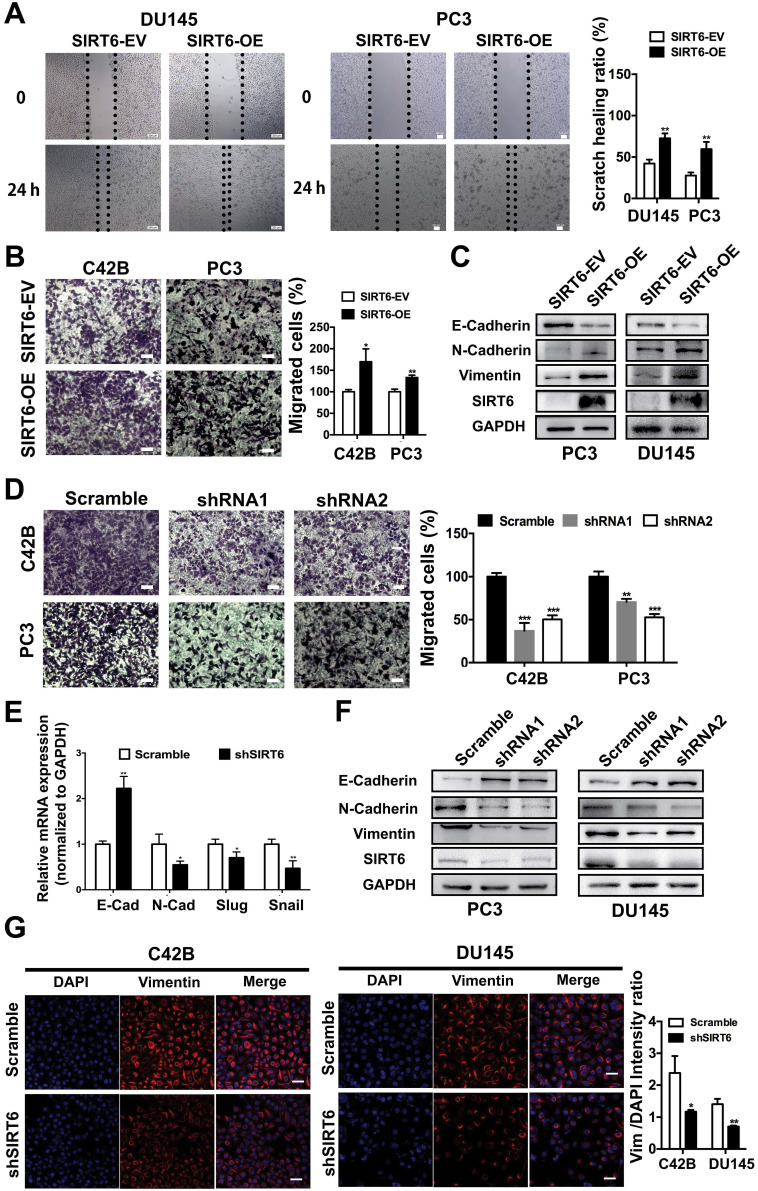
** SIRT6 induces a metastasis-promoting phenotype in prostate cancer cells. (A)** Wound healing assay and **(B)** migration assay of prostate cancer cell groups (stably overexpressing SIRT6, SIRT6-OE vs. empty vector control, SIRT6-EV). Scale bars, 100 µm. **(C)** Western blot of EMT related protein levels in SIRT6-EV cells and SIRT6-OE cells. **(D)** Migration assay of prostate cancer cell lines stably transfected with either empty vector (scramble) or shSIRT6. Scale bars, 100 µm. **(E-F)** The expression of EMT-related markers in these two groups of cells was analyzed by qPCR and Western blot. **(G)** Immunofluorescence of Vimentin in SIRT6 knockdown C42B cells (Vimentin, red; DAPI, blue nucleus; Scale bars, 50 µm).

The PC3M and C42B cell lines were metastatic and aggressive sublines derived from the PC3 and LNCap cell lines, respectively. The protein level of SIRT6 was slightly higher in aggressive prostate cell lines (PC3M and C42B) compared with the parental cell lines (PC3 and LNCap) (Figure S3A), indicating SIRT6 may affect metastatic phenotype of prostate cancer cells. The wound healing assay conducted in DU145 and PC3 cell lines revealed that overexpression of SIRT6 promoted the confluence of scratched cells (Figure 3A). In the transwell assay, the migration ability was significantly elevated in SIRT6-OE cells compared with SIRT6-EV cells (Figure 3B). Epithelial-mesenchymal transition (EMT) is an essential process in tumor metastasis, which facilitates cell migration and invasion 29, 30. Upregulation of SIRT6 inhibited the expression of epithelial marker E-cadherin and increased the expression of mesenchymal markers N-cadherin and Vimentin (Figure 3C, S3B). In consistence, knocking down SIRT6 significantly reduced the number of migrated cells compared to the scramble cells (Figure 3D). Meanwhile, silencing SIRT6 reversed the mesenchymal status as shown by higher E-cadherin and lower N-cadherin at both mRNA and protein levels (Figure 3E, 3F, S3C). Immunofluorescence staining also revealed that knocking down SIRT6 in C42B and DU145 cell lines remarkably repressed the expression of Vimentin, indicating an opposite process of EMT (Figure 3G). These findings together indicated that SIRT6 facilitated a metastasis promoting phenotype in prostate cancer cells.

### SIRT6 promotes the proliferation and metastasis of prostate cancer cells *in vivo*

**Figure 4:**
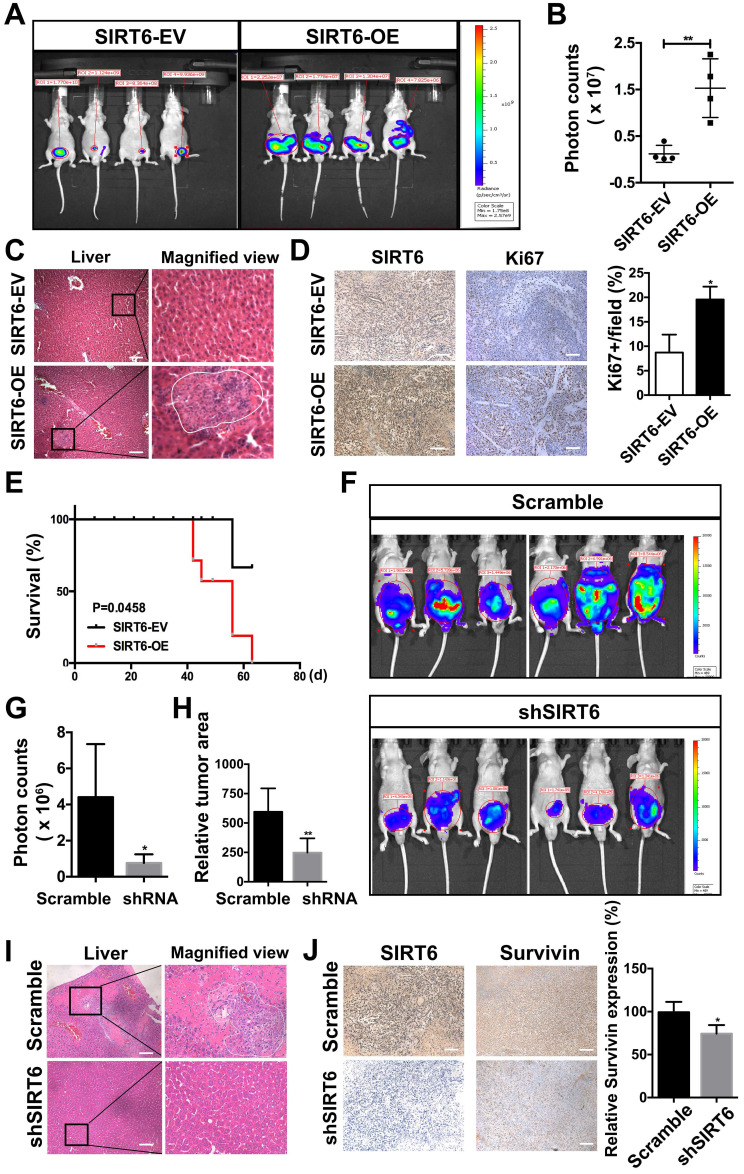
** SIRT6 promotes the proliferation and metastasis of prostate cancer cells *in vivo***. **(A)** PC3M-luc cells were stably transfected with control pCDH empty vector (SIRT6-EV) or pCDH SIRT6-overexpression vector (SIRT6-OE), and orthotopically implanted to BALC/c nude mice. The growth of orthotopically implanted tumor was monitored by bioluminescence, and quantification of luminescence was summarized in **(B). (C)** Representative images of HE staining (left panel) and metastasis in liver (right panel) of the SIRT6-overexpression orthotopic prostate cancer models compared with the control group. Scale bars, 100 µm.** (D)** IHC analysis of SIRT6 (left panel) and Ki67 (right panel) expression in tumors. Scale bars, 100 µm. **(E)** Survival of above-described two groups of mice: SIRT6-EV (n=6) vs. SIRT6-OE (n=6). **(F)** Bioluminescence imaging of orthotropic models from each group implanted with PC3M-luc cells stably transfected either with empty vector (scramble sequence) (n=6) or shSIRT6 vector (n=6). **(G)** Quantification and **(H)** area of luminescence in orthotropic models from each group.** (I)** Representative image of HE staining (left panel) and metastasis in liver (right panel) of the SIRT6 knockdown orthotopic prostate cancer model compared with the scramble group. Scale bars, 100 µm.** (J)** IHC analysis of SIRT6 (left panel) and Survivin (right panel) expression in tumors. Scale bars, 100 µm.

To evaluate the effect of SIRT6 on prostate cancer proliferation and metastasis *in vivo*, we needed to establish an orthotopic prostate cancer model. The highly aggressive, luciferase stably expressing PC3M cells (PC3M-luc) are widely used in metastatic orthotopic xenograft mouse model 31, 32. Then we developed the stable line of PC3M-luc cells to overexpress SIRT6 or silence SIRT6 expression. The *in vitro* experiments conducted in PC3M-luc also confirmed the tumor-promoting function of SIRT6 (Figure S4A, B). In SIRT6-overexpression orthotopic prostate cancer model, the tumor growth was measured by the bioluminescence (Figure 4A). A marked increase in tumor growth and metastasis was detected in PC3M-luc-SIRT6-OE group compared with the control group (Figure 4B, S4C). Consistently, more metastatic nodules were observed in the liver tissue section of the SIRT6-OE group (Figure 4C). The xenografted tumor sections from these two groups were processed to detect the proliferation rate of cells by immunofluorescence staining with an anti-Ki67 antibody, and increased cell proliferation rate in SIRT6-OE group was evident (Figure 4D). In addition, overexpression of SIRT6 worsened the survival status in tumor-bearing mice, accompanied by short overall survival and decreased body weight (Figure 4E, S4D). Furthermore, to test whether silencing SIRT6 could inhibit prostate cancer cell proliferation and metastatic ability *in vivo*, the SIRT6-knockdown PC3M-luc and control PC3M-luc cells were used to establish the orthotopic prostate cancer model. In accordance with previous results, silencing SIRT6 inhibited the malignance development with less metastasis area (Figure 4F, G, H). To confirm the bioluminescence result, mice were sacrificed for tissue collection, and the livers of the SIRT6 knockdown group showed absence or marked decrease of metastasis (Figure 4I). The tumor sections were processed to detect immunofluorescence staining with an anti-Survivin antibody, and the results indicated cell apoptosis rate in SIRT6-knockdown group was increased (Figure 4J). Additionally, silencing SIRT6 was associated with increased life span and stable body weight (Figure S4E, F). Collectively, these results further verified that SIRT6 could promote the proliferation and metastasis of prostate cancer both *in vitro* and *in vivo*.

### The tumor-promoting effect of SIRT6 involves multiple cancer-related signaling pathways, mainly the Notch pathway

**Figure 5:**
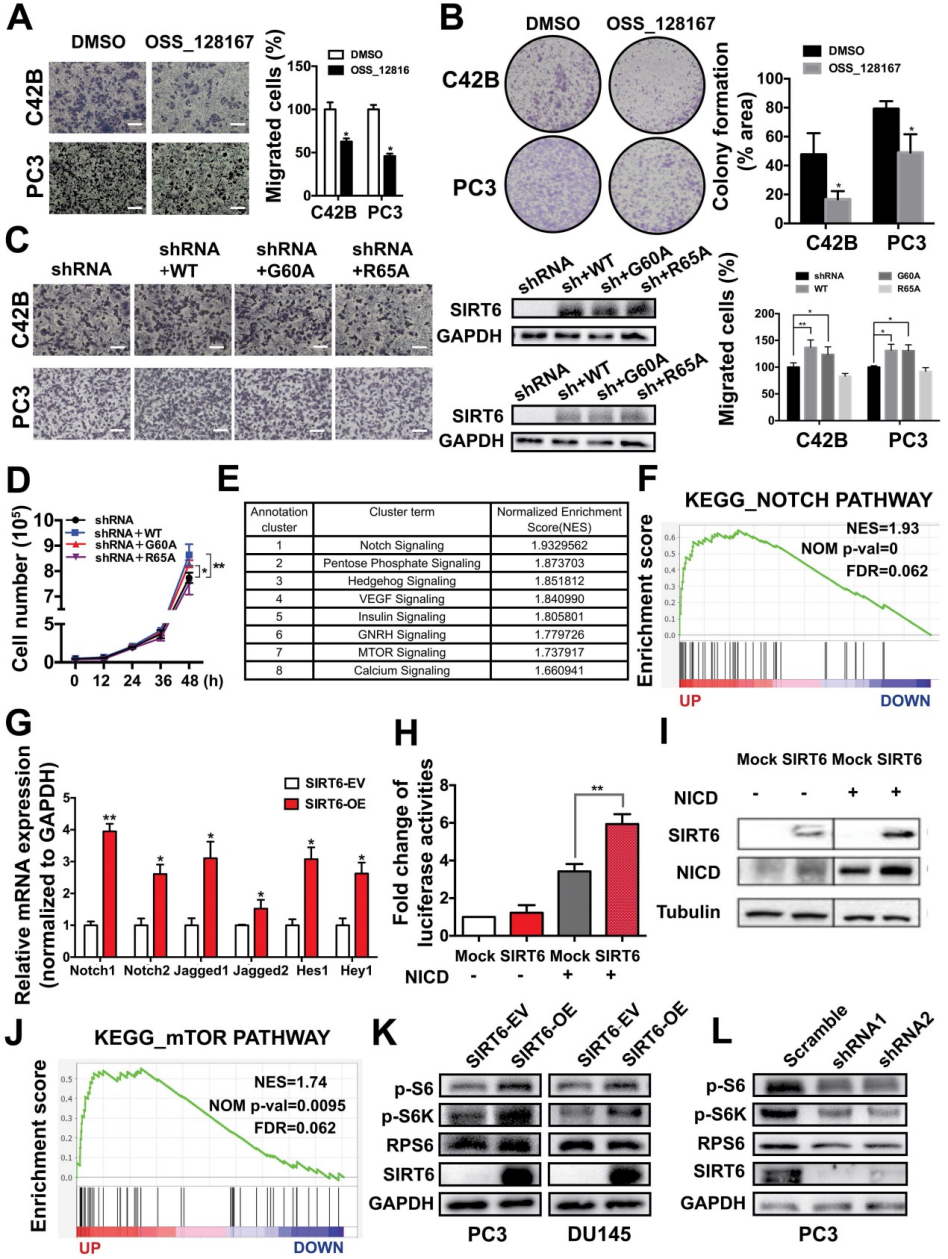
** The tumor-promoting effect of SIRT6 involves multiple cancer-related signaling pathways, mainly the Notch pathway. (A)** Migration assay and** (B)** colony formation of prostate cancer cells treated with SIRT6 deacetylation inhibitor OSS_128167. Scale bars, 100 µm. **(C)** Migration assay and **(D)** proliferation assay in SIRT6 knock-down prostate cancer cells transfected with SIRT6 WT or mutants (G60A and R65A). Scale bars, 100 µm.** (E)** Top 8 pathways significantly enriched in SIRT6 high expression group compared with low levels of SIRT6 group in Metastatic Prostate Cancer (SU2C/PCF Dream Team, cell 2015) dataset.** (F)** Enrichment plot of Notch pathway according to SIRT6 expression level in the same prostate cancer cohort. **(G)** The receptors and ligands expression of Notch pathway in stable SIRT6-OE cells and SIRT-EV cells determined by RT-PCR.** (H)** 4×CBF1 luciferase activity and **(I)** the expression of NICD in prostate cancer cells transfected with combination of NICD and SIRT6. **(J)** Enrichment plot of mTOR pathway according to SIRT6 expression level. **(K)** Overexpression of SIRT6 increases the levels of p-S6, p-S6K, downstream of mTOR pathway. **(L)** Silencing SIRT6 inhibits the expression of p-S6, p-S6K.

SIRT6 is a multifunctional enzyme possessing deacetylation, defatty acylation and ADP-ribosylation activities. The GSEA in Metastatic Prostate Cancer (SU2C/PCF Dream Team, Cell 2015) dataset from cBioportal found that histone deacetylation was enriched in the SIRT6 high expression group, indicating histone deacetylation may be associated with the malignancy development driven by SIRT6 (Figure S5A). To confirm this finding, we used the SIRT6 deacetylase inhibitor OSS_128167 33, 34. Migration abilities and colony formation were significantly inhibited by OSS_128167, which revealed that the deacetylation was involved in SIRT6-driven malignancy (Figure 5A, B). Moreover, SIRT6 R65A mutant and G60A mutant were utilized to identify whether the defatty-acylation and ADP-ribosylation of SIRT6 also contributed to tumor progression. The G60A mutant only maintains efficient defatty-acylation and the R65A mutant only shows ADP-ribosylation activity 35. We expressed the SIRT6 wild type and two SIRT6 mutants (G60A and R65A) in SIRT6 knock-down prostate cancer cells respectively and test their effect on proliferation and migration. The SIRT6 knock-down cells expressing SIRT6 G60A or SIRT6 WT showed similar increased cell proliferation and migration compared to SIRT6 knock-down cells (Figure 5C, D). In contrast, expressing the R65A mutant in SIRT6 knock-down cells had no significant effect (Figure 5C, D). Based on above results, both the deacetylation and defatty-acylation of SIRT6 were associated with the tumor-promoting effect. And the GSEA also showed that SIRT6 activated a variety of signaling pathways important in prostate cancer, among which the Notch pathway showed the highest Enrichment Score (Figure 5E, F). The results were confirmed by other prostate cancer cohort (Multi-Institute, Nat Med 2016) from cBioportal which also showed the co-expression of SIRT6 and Hes1/Hey1 (Figure S5B). Then we verified this finding in prostate cancer cell lines. The mRNA levels of the receptors and ligands in the Notch pathway were increased in the cells stably overexpressing SIRT6 (Figure 5G). Notch pathway activity was analyzed by assessing the signal of reporter gene 4×CBF1-luciferase. Overexpression of SIRT6 significantly enhanced reporter gene activity and NICD-1 expression (Figure 5H, 5I, S5C). And the reduced expression of Hes1 was observed in SIRT6 knockdown tumors tissues, indicating silencing SIRT6 also suppressed the Notch signaling pathway (Figure S5D). Furthermore, mTOR pathway was also markedly activated accompanied with SIRT6 high expression (Figure 5J). To our knowledge, this was the first report to associate SIRT6 and the mTOR pathway. We also verified this finding in prostate cancer cell lines that overexpression of SIRT6 increased the levels of p-S6, p-S6K, which were downstream of mTOR pathway (Figure 5K, S5E). Correspondingly, silencing SIRT6 suppressed the downstream molecules of mTOR pathway in both prostate cancer cell lines and *in vivo* tissue samples (Figure 5L, S5F, S5G).

### Preparation and characterization of E3-modified exosomes

**Figure 6:**
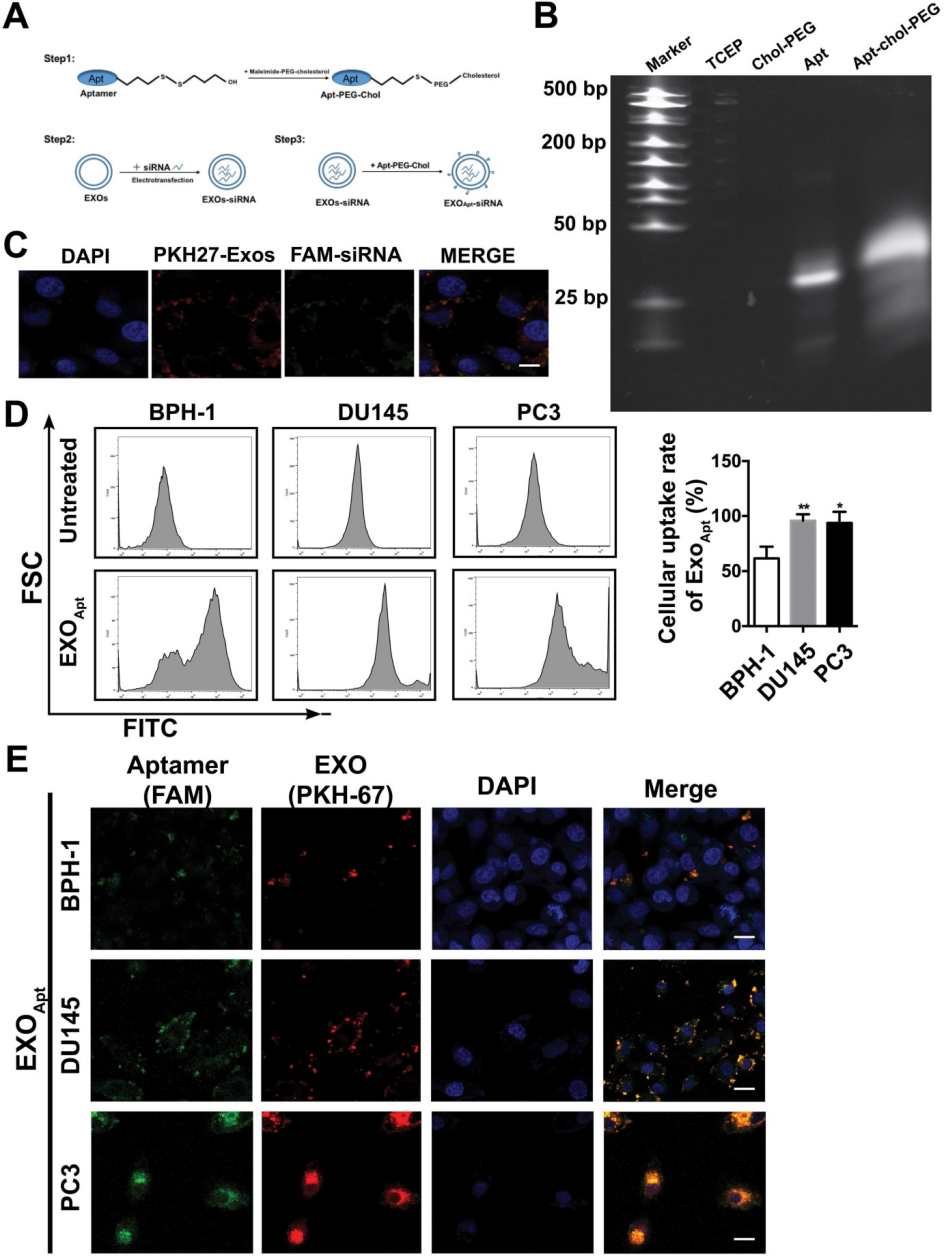
** Preparation and characterization of E3 aptamer-modified exosomes. (A)** Schematic illustration of the procedure for the E3 aptamer-modified siRNA loaded exosomes. **(B)** Gel electrophoresis of the Apt-PEG-Chol (Lane 5) compared to free E3 Apt (Lane 4). **(C)** FAM-labeled siRNA was loaded into PKH27-marked exosomes and applied to C42B cells. After 12 h incubation, cells were fixed and visualized by confocal microscopy (FAM-siRNA, green; PKH27-Exos, red; DAPI, blue for nucleus; Scale bars, 10 µm). **(D)** Flow cytometry analyses of the three cell lines after incubation with the E3 Apt-modified, DIO-labeled exosomes. **(E)** Confocal images of aptamer (FAM labeled)-modified exosomes (marked by PKH27) absorbed by three cell lines (FAM-Apt, green; PKH-27-Exos, red; DAPI, blue nucleus, scale bars, 20 µm).

Considering its significant role in prostate cancer progression, silencing SIRT6 in prostate cancer sites may be pharmacologically interesting, especially for the potential application in metastatic prostate cancer therapeutic strategies. Based on the result that both deacetylation and defatty acylation were responsible for the SIRT6-driven malignancy, we needed a specific SIRT6 inhibitor to suppress the two enzymatic activities, whereas the SIRT6 inhibitor OSS_128167 only inhibited the deacetylation. Thus, we used siRNA to assess whether targeting SIRT6 specifically might be therapeutically effective in prostate cancer. Moreover, a drug delivery system is needed in siRNA-mediated therapy due to the degradation of RNA 36. Exosomes are natural vesicles containing RNA, DNA, and proteins that can be absorbed by recipient cells to mediate biological processes. In recent years, a growing number of studies have applied exosomes to different types of diseases as drug delivery systems 37. Additionally, exosomes have shown better delivery efficiency and biocompatibility compared with artificial materials like liposomes 38, 39. Here, we utilized this novel and efficient drug delivery system to carry the siRNA to silence SIRT6 in targeted sites. This targeted delivery system was constructed stepwise (Figure 6A). First, exosomes were derived from HEK293T cells and isolated by ultracentrifugation. The purified exosomes were characterized as spherical particles by the negative staining TEM imaging (Figure S6A), which was in accordance with exosomes morphology as described earlier 40, and the presence of the exosomal markers was also verified by immunoblots (Figure S6B). To selectively target prostate cancer cells, we modified the surface of exosomes by attachment of an aptamer. The E3 aptamer was used because it could only be recognized and internalized by prostate cancer cells as reported previously 24. The first step is to link 3'-thiol modified aptamer and cholesterol-PEG-maleimide by thiol-maleimide cross-linking to synthesize the aptamer-PEG-cholesterol conjugate 27, 28, 41. To verify the conjugate, gel electrophoresis showed a slight increase in the molecular weight of the conjugate compared to the free aptamer, due to the attachment of the cholesterol-PEG 42 (Figure 6B). To test the efficacy of siRNA loading into exosomes by electroporation, the FAM-tagged siRNA was loaded into the PKH27-labeled exosomes, and applied to the recipient cell for 12 h. The fluorescence signals of the siRNA and exosomes could be both detected and were found to be co-localized (Figure 6C). Considering that cholesterol could insert into the cell membrane automatically, functionalization of exosomes was carried out by the co-incubation with exosomes in the presence of cholesterol-modified aptamer. To investigate the binding specificity and selectivity to prostate cancer, we employed three cell lines including prostate cancer cell line and non-cancerous prostate epithelial cell line to test the cellular uptake. Both DU145 cells and PC3 cells showed significantly higher mean fluorescent intensity compared with the non-cancerous epithelial BPH-1 cells as detected by flow cytometry (Figure 6D). These results demonstrated that the E3 aptamer-modified exosomes preferred binding to the prostate cancer cells instead of the noncancerous cells with higher selectivity and uptake efficiency. Furthermore, the cellular uptake was measured by confocal microscopy and the result was consistent with the data obtained from the flow cytometry (Figure 6E).

### Delivery of therapeutic SIRT6 siRNA by aptamer-modified exosomes suppresses tumor proliferation and metastasis

**Figure 7:**
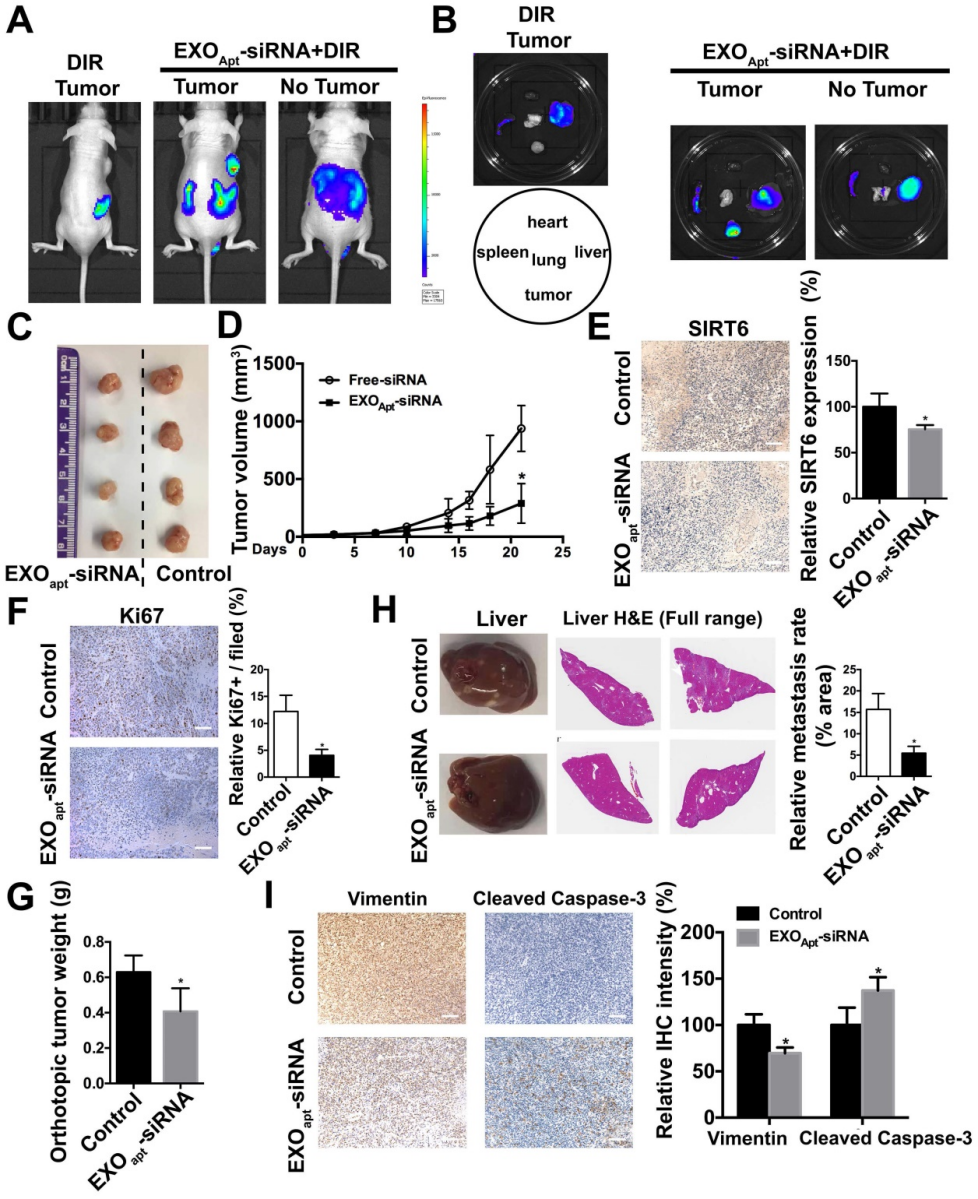
** Delivery of therapeutic SIRT6 siRNA by aptamer-modified exosomes suppresses prostate tumor proliferation and metastasis. (A)** Overall fluorescence imaging of tumor- bearing (subcutaneous implantation of PC3 cells) or no tumor-bearing mice after the injection of aptamer-modified, DIR-labeled exosomes. **(B)** *Ex vivo* fluorescence images of tumor and other major organs. **(C)** Representative image of the subcutaneous tumors in mice after treatment with aptamer-modified siRNA-loaded exosomes (n=4) compared with the control group (n=4). **(D)** Tumor volume changes of the subcutaneous mice models after treatment. **(E)** Representative IHC staining of SIRT6 in two groups of tumor tissues. Scale bars, 100 µm. **(F)** Representative IHC staining of Ki67 in two groups of tumor tissues. Scale bars, 100 µm. **(G)** The weight of tumor in orthotopically implanted mouse model after treatment with aptamer-modified siRNA-loaded exosomes (n=4) compared with the control group (n=4). **(H)** Full-scale HE staining of livers from the orthotopic mouse model. Metastatic nodules in livers were dissected counted. **(I)** Representative IHC staining of Vimentin and cleaved Caspase-3 in two groups of tumor tissues. Scale bars, 100 µm.

We assessed the targeting ability of E3 aptamer-modified exosomes to the tumor location in BALB/c mice bearing subcutaneously transplanted DU145 cells. The aptamer-modified, DIR-labeled exosomes were injected intravenously into the mice. Bioluminescence image showed that strong fluorescence signal was accumulated in the tumor area and liver (Figure 7A). The distribution of DIR fluorescence in different organs from the mice with or without tumor was shown in Figure 7B. Consistently, DIR fluorescence was strongest in tumors, but also found in the liver and spleen. All these results demonstrated that aptamer-modified exosomes showed potent targeting effect *in vivo*. To further determine the antitumor activity of aptamer-modified exosomes loaded with SIRT6 siRNA, we utilized the subcutaneous tumor model. Two weeks after implantation of DU145 tumor cells, mice were intravenously injected with aptamer-modified exosomes loaded with siRNA or the free-siRNA twice a week. The engineered exosomes containing siRNA evidently inhibited tumor growth (Figure 7C, 7D). The SIRT6 interfering efficiency in subcutaneous tumor model was confirmed by immunohistochemistry (Figure 7E) and the Ki67 staining of tumor tissues showed that the engineered exosomes suppressed the prostate cancer cell proliferation (Figure 7F). Treatment with engineered exosomes also resulted in an antitumor effect compared with the control group, accompanied with stable body weight (Figure S7A). Next, we tested the therapeutic efficacy in orthotopic prostate tumor model by using PC3 cell line. Treatment with engineered exosomes reduced orthotopic tumor growth (Figure 7G) and liver metastatic burden (Figure 7H). The SIRT6 interfering efficiency in orthotopic transplantation tumor models was confirmed by immunohistochemistry (Figure S7B). Additionally, the treated orthotopic xenografts showed decreased expression of Vimentin and elevated expression of cleaved Capase-3 compared with the control group. Together, targeted inhibition of SIRT6 via engineered exosomes impaired the proliferation and metastasis in prostate cancer.

## Discussion

SIRT6 plays dual roles in cancer as both a tumor suppressor and an oncogene with tissue-specific pattern 43. Initially, SIRT6 was regarded to be a tumor suppressor in a variety of cancer types by controlling cancer metabolism 12, 44, 45. Recently, emerging studies have uncovered the tumor-promoting function of SIRT6. For example, SIRT6 is shown to be a driver gene in the skin cancer by promoting survival and proliferation 15. Overexpression of SIRT6 in breast cancer cells increased the resistance to anticancer drugs 46. However, little has been identified about the detailed function of SIRT6 in prostate cancer. Here, our study implicates the pro-metastatic role of SIRT6 in prostate cancer and uncovers the mechanism involved in malignant progression driven by SIRT6. As a follow-up study to our previous report that silencing SIRT6 reduced the prostate cancer cell viability, this work has several novel findings. First, we found for the first time that SIRT6 accelerated the metastasis in prostate cancer. Overexpression or knockdown of SIRT6 in prostate cancer cells significantly promoted or inhibited migration both *in vitro* and *in vivo*. Second, we identified the downstream signals of SIRT6 in prostate cancer by GSEA, revealing a significant enrichment of SIRT6-induced signaling pathway activation signature. Finally, we used exosomes modified with a specific aptamer as an efficient siRNA delivery system, which targeted the prostate cancer cells only to silence SIRT6 *in vivo*. Targeting SIRT6 could serve as a promising method for advanced prostate cancer instead of the traditional drugs.

Owing to the multiple catalytic function and a broad substrate spectrum, SIRT6 seems to promote prostate cancer progression in a diverse and complex way. GSEA reveals that SIRT6 caused activation of various oncogenic pathways, which contributed to its malignancy. According to the Enrichment Score, Notch pathway was the most relevant pathway involved in SIRT6-dependent mechanism. The Notch pathway is an evolutionarily conserved signaling pathway that regulates the differentiation, cell-fate determination in both embryonic and adult organs 47, 48. Accumulating evidence has demonstrated Notch signaling facilitates prostatic tumorigenesis and metastasis, which is proposed to be potential therapeutic targets 49-51. For instance, chronically activated Notch 1 receptor and jagged-1 ligand both accelerated progression and metastasis of prostate cancer 52, 53. The GSEA result was verified in prostate cancer cell lines that overexpression of SIRT6 could activate the Notch pathway by upregulating the expression of Notch receptors and ligands. Additionally, overexpression of SIRT6 significantly enhanced reporter gene activity and the nuclear import of NICD *in vitro*. Thus, the pro-tumorigenic role of SIRT6 is likely to be mediated by activation of the Notch pathway. In contrast to a previous report in which SIRT6 inhibited the Notch pathway by significantly reducing the level of H3K9ac in the promoter region of Notch1 and Notch4 in podocyte injury 54, we found that SIRT6 activated the Notch pathway in prostate cancer, indicating that a different molecular mechanism of the activation of Notch pathway by SIRT6 might exist in prostate cancer. In most cases, diverse functions of SIRT6 are carried out by its deacetylation activity of H3K9 and H3K56 to suppress the transcription and inhibit downstream pathways. Apart from histone deacetylation, SIRT6 can also directly deacetylate other proteins to regulate their stability and activity 11. Thus, we speculate that SIRT6 activate the Notch pathway by directly modifying key components in the pathway and this may be a critical next step for further study to elucidate the relationship of Notch pathway and SIRT6.

Small interfering RNA is a powerful gene silencing tool to selective suppression of specific genes of interest, showing great potential in gene therapy 55-57. To achieve the successful application of siRNA *in vivo*, a safe siRNA delivery system with high efficacy is needed to protect siRNA from degradation and direct siRNA specifically to prostate cancer cells. Due to the intrinsic roles to transport small RNA molecules between cells, exosomes have been regarded as a viable and nontoxic vehicle for therapeutic nucleic acids delivery applications 16, 17. Further, to confer the exosomes with the cancer targeting capacity, tumor-selective aptamers can be modified on the membrane of exosomes to have high binding affinity with tumor cells and even favorable penetration within the tumor. The E3 aptamer has been recently identified that could be recognized by prostate cancer cells rather than normal prostate cells 24. Based on above reasons, we conjugated the E3 aptamer on the surface of exosomes and loaded the engineered exosomes with siRNA by electroporation. Our results showed that both the subcutaneous tumor model and the orthotopic tumor model responded well to the engineered exosomes. In this respect, targeting SIRT6 in prostate cancer with engineered exosomes simulates the potential clinical therapeutic efficiency, resulting in lower proliferation rate of tumor and less metastasis area.

In conclusion, SIRT6 is a novel and promising target in metastatic castration-resistant prostate cancer. The aptamer-modified exosomes to silence SIRT6 expression may provide some first hints for clinical translation.

## Supplementary Material

Supplementary figures.

## Abbreviations

AR: androgen receptor
ADT: androgen deprivation therapy
mCRPC: metastatic castration-resistant prostate cancer
GSEA: gene set enrichment analysis
IHC: immunohistochemistry
IF: immunofluorescence microscopy
SIRT6-OE: SIRT6 overexpressing vector
SIRT6-EV: SIRT6 empty vector

## References

[1] Culp MB, Soerjomataram I, Efstathiou JA, Bray F, Jemal A (2020). Recent global patterns in prostate cancer incidence and mortality rates. Eur Urol.

[2] Davies A, Conteduca V, Zoubeidi A, Beltran H (2019). Biological evolution of castration-resistant prostate cancer. Eur Urol Focus.

[3] Karantanos T, Corn PG, Thompson TC (2013). Prostate cancer progression after androgen deprivation therapy: mechanisms of castrate resistance and novel therapeutic approaches. Oncogene.

[4] Zong Y, Goldstein AS (2013). Adaptation or selection - mechanisms of castration-resistant prostate cancer. Nat Rev Urol.

[5] Finkel T, Deng CX, Mostoslavsky R (2009). Recent progress in the biology and physiology of Sirtuins. Nature.

[6] Hiroyasu Y, Kristina S, Johan A (2007). Sirtuin functions in health and disease. Mol Endocrinol.

[7] Michan S, Sinclair D (2007). Sirtuins in mammals: insights into their biological function. Biochem J.

[8] Kawahara TL, Michishita E, Adler AS, Damian M, Berber E, Lin M (2009). SIRT6 links histone H3 Lysine 9 deacetylation to NF-κB-Dependent gene expression and organismal life span. Cell.

[9] Wang H, Diao D, Shi Z, Zhu X, Gao Y, Gao S (2016). SIRT6 controls hematopoietic stem Cell homeostasis through epigenetic regulation of Wnt signaling. Cell Stem Cell.

[10] Yang Q, Hu J, Yang Y, Chen Z, Feng J, Zhu Z (2020). Sirt6 deficiency aggravates angiotensin II-induced cholesterol accumulation and injury in podocytes. Theranostics.

[11] Kugel S, Mostoslavsky R (2014). Chromatin and beyond: the multitasking roles for SIRT6. Trends Biochem Sci.

[12] Sebastián C, Zwaans BM, Silberman DM, Gymrek M, Goren A, Zhong L (2012). The histone deacetylase SIRT6 is a tumor suppressor that controls cancer metabolism. Cell.

[13] Liu Y, Xie QR, Wang B, Shao J, Zhang T, Liu T (2013). Inhibition of SIRT6 in prostate cancer reduces cell viability and increases sensitivity to chemotherapeutics. Protein Cell.

[14] Bauer I, Grozio A, Lasigliè D, Basile G, Sturla L, Magnone M (2012). The NAD+-dependent histone deacetylase SIRT6 promotes cytokine production and migration in pancreatic cancer cells by regulating Ca2+ responses. J Biol Chem.

[15] Ming M, Han W, Zhao B, Sundaresan NR, Deng CX, Gupta MP (2014). SIRT6 promotes COX-2 expression and acts as an oncogene in skin cancer. Cancer Res.

[16] Xu R, Rai A, Chen M, Suwakulsiri W, Greening DW, Simpson RJ (2018). Extracellular vesicles in cancer - implications for future improvements in cancer care. Nat Rev Clin Oncol.

[17] Zhang ZG, Buller B, Chopp M (2019). Exosomes - beyond stem cells for restorative therapy in stroke and neurological injury. Nat Rev Neurol.

[18] Théry C, Zitvogel L, Amigorena S (2002). Exosomes: composition, biogenesis and function. Nat Rev Immunol.

[19] Haney MJ, Klyachko NL, Zhao Y, Gupta R, Plotnikova EG, He Z (2015). Exosomes as drug delivery vehicles for Parkinson's disease therapy. J Control Release.

[20] Tian Y, Li S, Song J, Ji T, Zhu M, Anderson GJ (2014). A doxorubicin delivery platform using engineered natural membrane vesicle exosomes for targeted tumor therapy. Biomaterials.

[21] Safdar A, Saleem A, Tarnopolsky MA (2016). The potential of endurance exercise-derived exosomes to treat metabolic diseases. Nat Rev Endocrinol.

[22] Luo ZW, Li FX, Liu YW, Rao SS, Yin H, Huang J (2019). Aptamer-functionalized exosomes from bone marrow stromal cells target bone to promote bone regeneration. Nanoscale.

[23] Li Y, Gao Y, Gong C, Wang Z, Xia Q, Gu F (2018). A33 antibody-functionalized exosomes for targeted delivery of doxorubicin against colorectal cancer. Nanomedicine.

[24] Powell Gray B, Kelly L, Ahrens DP, Barry AP, Kratschmer C, Levy M (2018). Tunable cytotoxic aptamer-drug conjugates for the treatment of prostate cancer. Proc Natl Acad Sci U S A.

[25] Quan Y, Wang N, Chen Q, Xu J, Cheng W, Di M (2015). SIRT3 inhibits prostate cancer by destabilizing oncoprotein c-MYC through regulation of the PI3K/Akt pathway. Oncotarget.

[26] El-Andaloussi S, Lee Y, Lakhal-Littleton S, Li J, Seow Y, Gardiner C (2012). Exosome-mediated delivery of siRNA *in vitro* and *in vivo*. Nat Protoc.

[27] Alshaer W, Hillaireau H, Vergnaud J, Ismail S, Fattal E (2015). Functionalizing liposomes with anti-CD44 aptamer for selective targeting of cancer cells. Bioconjug Chem.

[28] Wan Y, Wang L, Zhu C, Zheng Q, Wang G, Tong J (2018). Aptamer-conjugated extracellular nanovesicles for targeted drug delivery. Cancer Res.

[29] Tsuji T, Ibaragi S, Hu GF (2009). Epithelial-mesenchymal transition and cell cooperativity in metastasis. Cancer Res.

[30] Tsai JH, Yang J (2013). Epithelial-mesenchymal plasticity in carcinoma metastasis. Genes Dev.

[31] Jenkins DE, Yu SF, Hornig YS, Purchio T, Contag PR (2003). *In vivo* monitoring of tumor relapse and metastasis using bioluminescent PC-3M-luc-C6 cells in murine models of human prostate cancer. Clin Exp Metastasis.

[32] Uysal-Onganer P, Djamgoz MB (2007). Epidermal growth factor potentiates *in vitro* metastatic behaviour of human prostate cancer PC-3M cells: involvement of voltage-gated sodium channel. Mol Cancer.

[33] Jiang H, Cheng ST, Ren JH, Ren F, Yu HB, Wang Q (2019). SIRT6 inhibitor, OSS_128167 restricts hepatitis B virus transcription and replication through targeting transcription factor peroxisome proliferator-activated receptors α. Front Pharmacol.

[34] Yang J, Zhou X, Li Y, Zhang Y, Wang X (2019). Targeting Sirt6 with OSS_128167 displays anti-tumor activities in diffuse large B-Cell lymphoma through down-regulation of PI3K Signaling. Blood.

[35] Zhang X, Khan S, Jiang H, Antonyak MA, Chen X, Spiegelman NA (2016). Identifying the functional contribution of the defatty-acylase activity of SIRT6. Nat Chem Biol.

[36] Oh YK, Park TG (2009). siRNA delivery systems for cancer treatment. Adv Drug Deliv Rev.

[37] Batrakova EV, Kim MS (2015). Using exosomes, naturally-equipped nanocarriers, for drug delivery. J Control Release.

[38] Stremersch S, Vandenbroucke RE, Van Wonterghem E, Hendrix A, De Smedt SC, Raemdonck K (2016). Comparing exosome-like vesicles with liposomes for the functional cellular delivery of small RNAs. J Control Release.

[39] Kamerkar S, LeBleu VS, Sugimoto H, Yang S, Ruivo CF, Melo SA (2017). Exosomes facilitate therapeutic targeting of oncogenic KRAS in pancreatic cancer. Nature.

[40] Théry C, Amigorena S, Raposo G, Clayton A (2006). Isolation and characterization of exosomes from cell culture supernatants and biological fluids. Curr Protoc Cell Biol.

[41] Wan Y, Wang L, Zhu C, Zheng Q, Wang G, Tong J (2018). Aptamer-conjugated extracellular nanovesicles for targeted drug delivery. Cancer Res.

[42] Alshaer W, Hillaireau H, Vergnaud J, Mura S, Deloménie C, Sauvage F (2018). Aptamer-guided siRNA-loaded nanomedicines for systemic gene silencing in CD-44 expressing murine triple-negative breast cancer model. J Control Release.

[43] Lerrer B, Gertler AA, Cohen HY (2016). The complex role of SIRT6 in carcinogenesis. Carcinogenesis.

[44] Lyssiotis CA, Cantley LC (2012). SIRT6 puts cancer metabolism in the driver's seat. Cell.

[45] Zhang Y, Nie L, Xu K, Fu Y, Zhong J, Gu K, Zhang L (2019). SIRT6, a novel direct transcriptional target of FoxO3a, mediates colon cancer therapy. Theranostics.

[46] Khongkow M, Olmos Y, Gong C, Gomes AR, Monteiro LJ, Yagüe E (2013). SIRT6 modulates paclitaxel and epirubicin resistance and survival in breast cancer. Carcinogenesis.

[47] Bray SJ (2006). Notch signalling: a simple pathway becomes complex. Nat Rev Mol Cell Biol.

[48] Louvi A, Artavanis-Tsakonas S (2006). Notch signalling in vertebrate neural development. Nat Rev Neurosci.

[49] Leong KG, Gao WQ (2008). The Notch pathway in prostate development and cancer. Differentiation.

[50] Marignol L, Rivera-Figueroa K, Lynch T, Hollywood D (2013). Hypoxia, notch signalling, and prostate cancer. Nat Rev Urol.

[51] Marignol L (2014). Targeting notch in prostate cancer-combination is the key. Nat Rev Urol.

[52] Yu H, Li S, Wang Z, Ding W, Zhang Y (2008). Notch1 regulates the expression of its ligand Jagged1 in PC3 prostate cancer cells. Clin Cancer Res.

[53] Zhu H, Zhou X, Redfield S, Lewin J, Miele L (2013). Elevated Jagged-1 and Notch-1 expression in high grade and metastatic prostate cancers. Am J Transl Res.

[54] Liu M, Liang K, Zhen J, Zhou M, Wang X, Wang Z (2017). Sirt6 deficiency exacerbates podocyte injury and proteinuria through targeting Notch signaling. Nat Commun.

[55] Burnett JC, Rossi JJ, Tiemann K (2011). Current progress of siRNA/shRNA therapeutics in clinical trials. Biotechnol J.

[56] Caplen NJ (2003). RNAi as a gene therapy approach. Expert Opin Biol Ther.

[57] Kaur IP, Chopra K, Rishi P, Puri S, Sharma G (2014). Small RNAs: the qualified candidates for gene manipulation in diverse clinical pathologies. Crit Rev Ther Drug Carrier Syst.

