# Supplementary material for: Targeted inhibition of SIRT6 via engineered exosomes impairs tumorigenesis and metastasis in prostate cancer
Source: Theranostics. 2021 Apr 26;11(13):6526–41. doi: 10.7150/thno.53886 (PMC8120217; doi:10.7150/thno.53886)
Supplement: Supplementary file 1 — Supplementary figures. [file thnov11p6526s1.pdf]

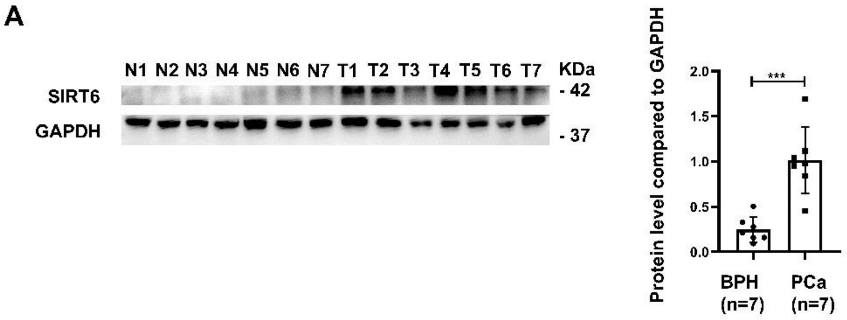

Supplementary Figure 1. The protein level of SIRT6 in prostate cancer tissues. A. High expression level of SIRT6 in prostate cancer tissues (T1-T7) compared with human prostate tissues (N1-N7).

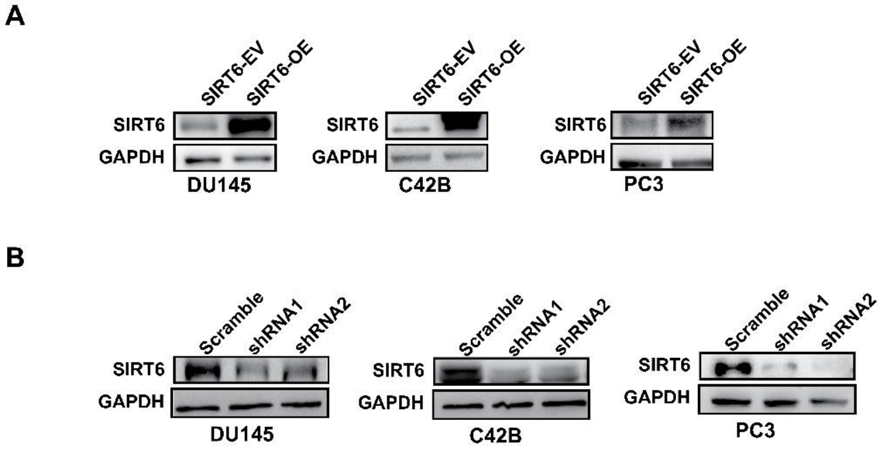

Supplementary Figure 2. Overexpression and knockdown of SIRT6 in prostate cancer cell lines. A. Western blot analysis of SIRT6 levels in stably overexpressed (SIRT6-OE) and control (SIRT6-EV) cells. **B.** Western blot analysis of SIRT6 levels in two stably knockdown (shRNA1, shRNA2) and control (Scramble) cells.

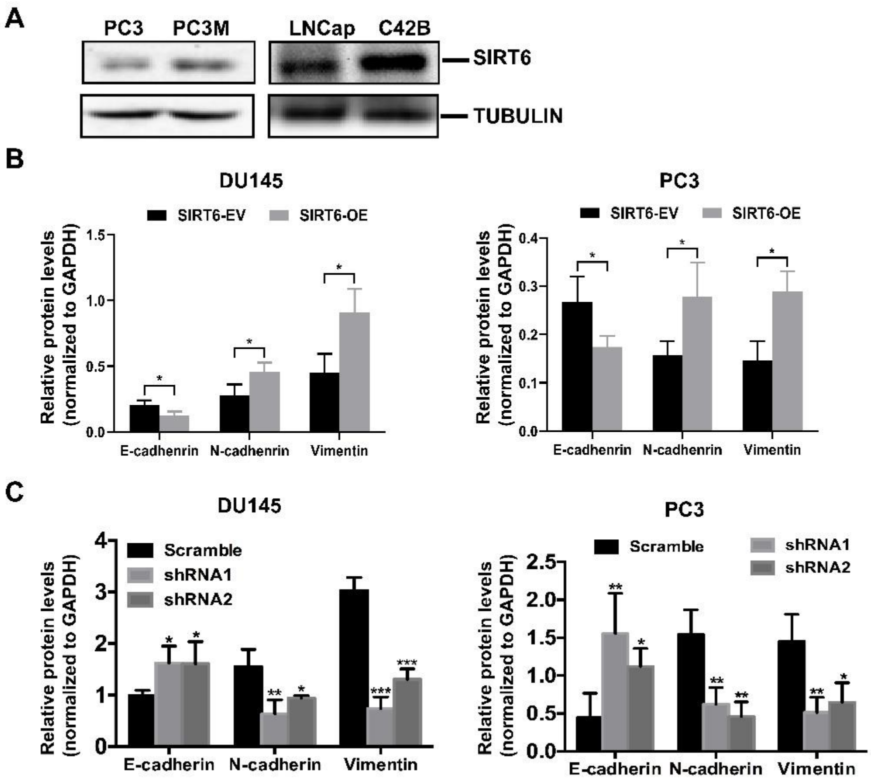

Supplementary Figure 3. SIRT6 promotes EMT in prostate cancer cells. A. Western blot analysis of SIRT6 expression in parental prostate cell lines (PC3 and LNCap) and the derived metastatic prostate cell lines (PC3M and C42B). **B-C.** Quantitative analysis of the expression of EMT markers in different prostate cancer cells with SIRT6 being either up- or down-regulated.

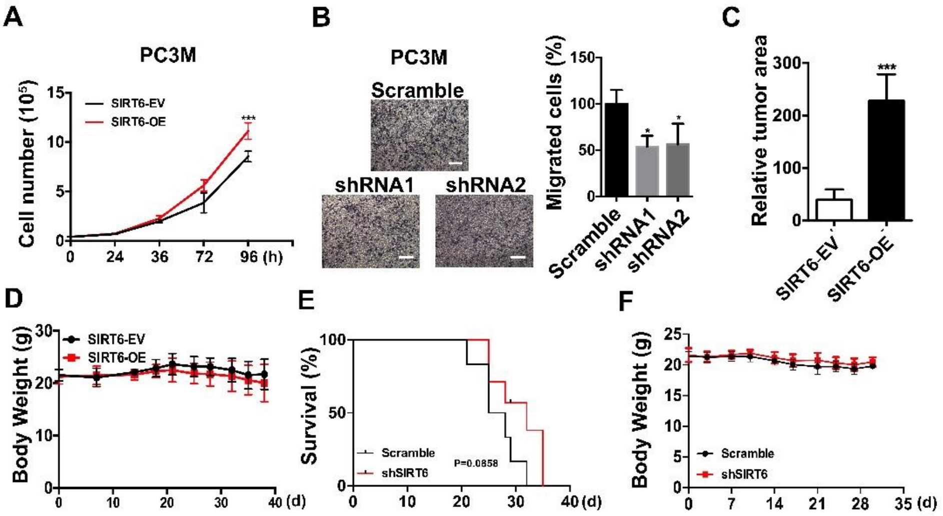

Supplementary Figure 4. SIRT6 regulates prostate cancer cells metastasis in vivo. A. Proliferation assay of SIRT6 stably overexpressed and control prostate cancer cells. **B.** Migration assay of SIRT6 stably knockdown and control prostate cancer cells. **C.** Relative area of luminescence in orthotopic mice models (PC3M-luc cells stably transfected with either control pCDH empty vector, SIRT6-EV or pCDH SIRT6-overexpression vector, SIRT6-OE). **D.** Body weight of orthotopic mice models (SIRT6-EV vs. SIRT6-OE). **E-F.** Survival and body weight of orthotopic mice models (PC3M-luc cells stably transfected with either SIRT6-Scramble or SIRT6-shRNA).

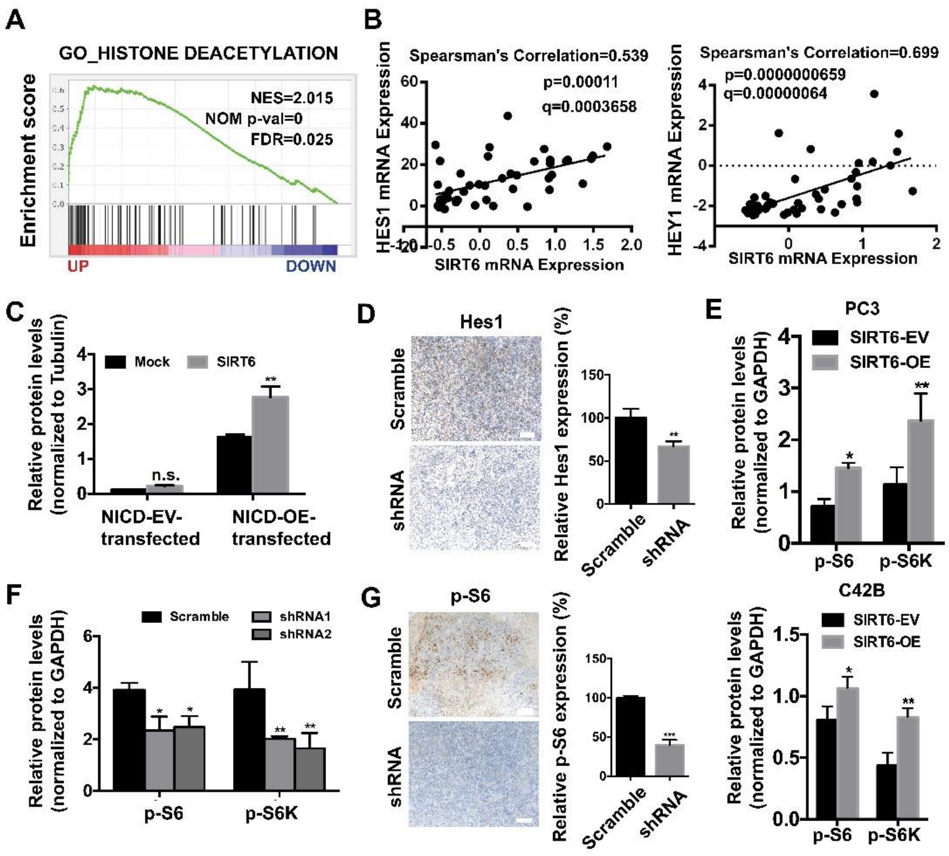

Supplementary Figure 5. The tumor promoting effect of SIRT6 involves multiple cancer-related signaling. A. Histone deacetylation is significantly enriched in SIRT6 high expression group. **B.** Correlation between SIRT6 and the downstream genes of the Notch pathway, Hes1 and Hey1 in prostate cancer cohort (Multi-Institute, Nat Med 2016) dataset. **C.** Quantitative analysis of NICD levels in PC3 prostate cancer cell (co-transfected with combinations of NICD-overexpression vector, SIRT6-overexpression vector, and their corresponding control vectors). **D.** Representative IHC staining of Hes1 in two groups of tumor tissues. **E-F.** Quantitative analysis of the levels of p-S6/p-S6K in different prostate cancer cells with SIRT6 being up- or down-regulated. **G.** Representative IHC staining of p-S6 in two groups of tumor tissues.

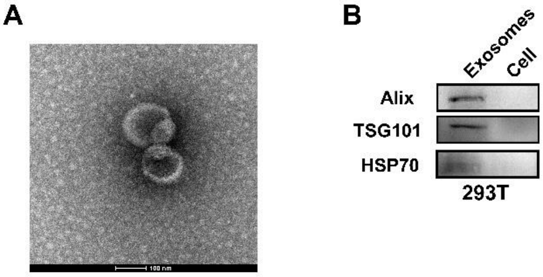

Supplementary Figure 6. The characterization of exosomes derived from 293T cells. A. TEM imaging of 293T-derived exosomes. **B.** Western blot analysis of exosomal protein markers.

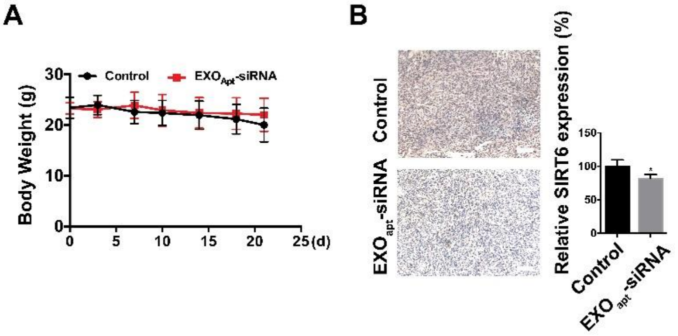

Supplementary Figure 7. Therapeutic SIRT6 siRNA delivery by aptamer-modified exosomes suppresses tumor proliferation and metastasis. A. Body weight changes of orthotopic mouse model after treatment of aptamer-modified siRNA-loaded exosomes vs. the control group. **B.** Representative IHC staining of SIRT6 in orthotopically implanted tumor tissues.
